# MAC: identifying and correcting annotation for multi-nucleotide variations

**DOI:** 10.1186/s12864-015-1779-7

**Published:** 2015-08-01

**Authors:** Lei Wei, Lu T. Liu, Jacob R. Conroy, Qiang Hu, Jeffrey M. Conroy, Carl D. Morrison, Candace S. Johnson, Jianmin Wang, Song Liu

**Affiliations:** Department of Biostatistics & Bioinformatics, Roswell Park Cancer Institute, Buffalo, NY USA; Center for Personalized Medicine, Roswell Park Cancer Institute, Buffalo, NY USA; Department of Pharmacology and Therapeutics, Roswell Park Cancer Institute, Buffalo, NY USA

## Abstract

**Background:**

Next-Generation Sequencing (NGS) technologies have rapidly advanced our understanding of human variation in cancer. To accurately translate the raw sequencing data into practical knowledge, annotation tools, algorithms and pipelines must be developed that keep pace with the rapidly evolving technology. Currently, a challenge exists in accurately annotating multi-nucleotide variants (MNVs). These tandem substitutions, when affecting multiple nucleotides within a single protein codon of a gene, result in a translated amino acid involving all nucleotides in that codon. Most existing variant callers report a MNV as individual single-nucleotide variants (SNVs), often resulting in multiple triplet codon sequences and incorrect amino acid predictions. To correct potentially misannotated MNVs among reported SNVs, a primary challenge resides in haplotype phasing which is to determine whether the neighboring SNVs are co-located on the same chromosome.

**Results:**

Here we describe MAC (Multi-Nucleotide Variant Annotation Corrector), an integrative pipeline developed to correct potentially mis-annotated MNVs. MAC was designed as an application that only requires a SNV file and the matching BAM file as data inputs. Using an example data set containing 3024 SNVs and the corresponding whole-genome sequencing BAM files, we show that MAC identified eight potentially mis-annotated SNVs, and accurately updated the amino acid predictions for seven of the variant calls.

**Conclusions:**

MAC can identify and correct amino acid predictions that result from MNVs affecting multiple nucleotides within a single protein codon, which cannot be handled by most existing SNV-based variant pipelines. The MAC software is freely available and represents a useful tool for the accurate translation of genomic sequence to protein function.

## Background

As the use of Next-Generation Sequencing (NGS) technologies for studying human disease continues to grow, defects in the cancer genome and their association with disease progression and treatment options are being reported at an astonishing rate. Single nucleotide variants (SNVs), one of the most prevalent mutation types, often occur in cancer related genes and can result in amino acid changes and nonfunctional proteins. Accurate prediction of any possible downstream consequences to the amino acid residue is one critical analytical requirement for any robust NGS pipeline. Without accurate variant annotation, further exploitation of the NGS data in both research and clinical contexts can be compromised [1].

**Fig. 1:**
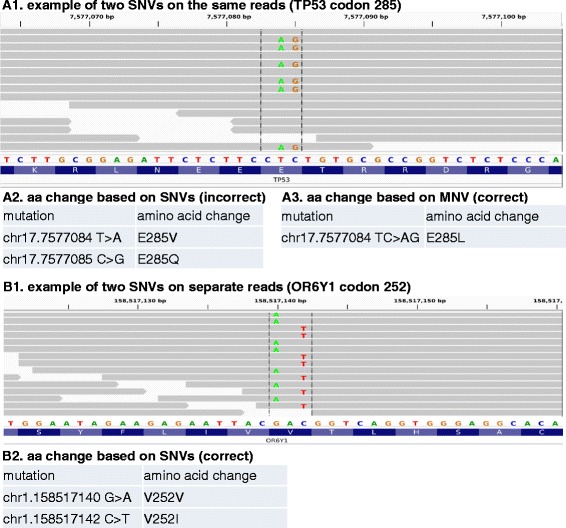
Amino acid predictions for two neighboring SNVs scenarios. (A1) Two consecutive SNVs in gene TP53 codon 285. The fact the two SNVs are present on the same read suggests they are originated from the same chromosome. (A2) Incorrect annotation based on prediction of individual SNVs. The first and second SNVs were predicted to introduce E285V and E285Q, respectively. (A3) The correct amino acid change based on MNV is E285L. (B1) Two SNVs are located in gene OR6Y1 codon 252 but on different reads, suggesting they originated from separate chromosomes. (B2) The two SNVs in B1 were correctly predicted to introduce V252V and V252I based on individual SNVs. The sequencing reads are displayed in IGV viewer [14]

It has been observed that certain cancers present a signature pattern of MNVs where substitution mutations occur at consecutive bases [2]. This phenomenon may reflect pathological history such as tobacco usage or ultraviolet light exposure [3–6]. Although MNVs can be identified by common variant callers, development of corresponding annotation tools has been lagging behind. Most existing variant callers report a MNV as individual SNVs [7, 8]. As a result, the predicted amino acid change is likely incorrect when a MNV occurs at multiple bases within the same protein codon (Fig. 1 A1-3). Existing annotators can predict the amino acid change for MNVs, but must rely on user-provided, mostly manually curated, MNV data sources. For mutations automatically called by SNV-based pipelines, there is a pressing need for automated tools to identify and fix any incorrectly annotated MNVs.

The challenge in distinguishing a MNV from neighboring SNVs from separate chromosomes resides in haplotype phasing and the ability to determine the combination of alleles found on the same chromosome (Fig. 1, A1 vs B1). Retrieving haplotype information from NGS reads is not entirely new, but most published methods specialize in germline polymorphisms and typically rely on population genetics information [9]. To date there has been no published program to identify MNVs from existing SNV calls and to restore incorrectly predicted amino acids.

Here we present MAC, a software designed to automatically correct MNV annotation generated from any existing SNV-based variant pipeline. By processing a list of previously detected SNVs and the corresponding raw data in Sequence Alignment/Map (SAM/BAM) format [8], MAC builds a multigraph containing SNVs with haplotype phase and codon information to identify connected components defined as a *Block of Mutation within Codon* (BMC), a structural unit containing a potentially mis-annotated amino acid. For every BMC, MAC further extracts every existing haplotype and annotates it using a user-specified variant annotator. For convenience, we have precompiled MAC to work with three popular annotators: ANNOVAR, SnpEff and VEP [10–12]. To be flexible with other annotators, MAC also provides a ‘no-annotation’ mode. Under this mode, MAC will output raw haplotype phase information which can be used as the input for any user preferred annotation tool. The accessibility of this tool, together with its flexibility and robustness, should facilitate accurate annotation of these infrequently occurring but potentially significant variant subtypes.

## Implementation

MAC is implemented in Perl and can be run on any Linux/Unix-like environment with installation of Perl and Bio-SamTools package. An overview of the MAC pipeline is provided below.

### Input

MAC requires (i) a list of previously called SNVs and (ii) the corresponding BAM file. To be flexible with all existing pipelines, the SNV list can be generated from any caller and only basic information is required: mutan/mutant alleles.

### Selection of the annotator by the user

**Fig. 2:**
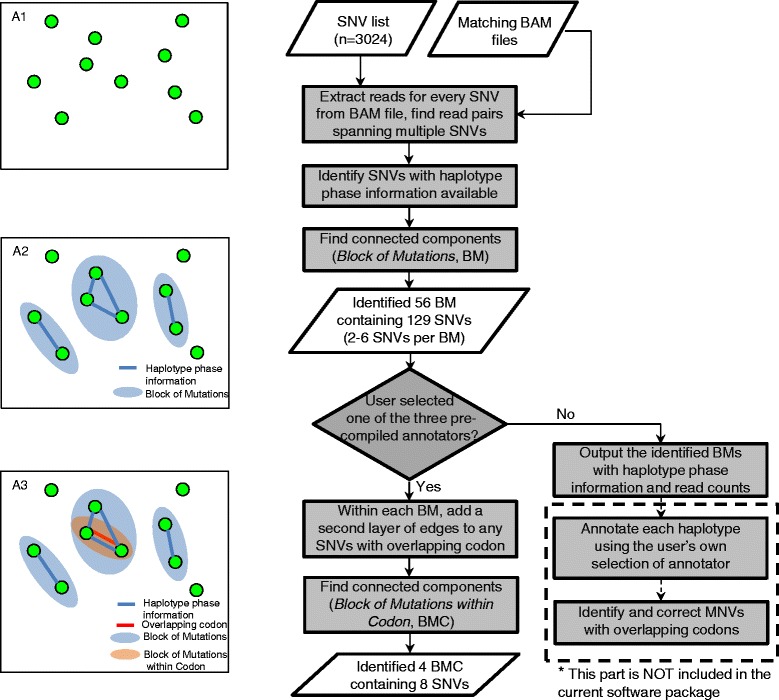
Depiction of MAC workflow (left panel) and a MAC test run (*right* panel). *Left*: (A1) A list of SNVs identified by any variant caller; (A2) Reads extracted from the BAM file for all SNVs to identify *Block of Mutations*; (A3) Identify *Block of Mutations within Codon* within each subgraph using an annotation tool. *Right*: MAC test run using 3024 input SNVs from a breast cancer data set identified 56 BMs and 4 BMCs containing 8 SNVs. After re-annotation, 7 of 8 SNVs were classified as MNVs with different amino acid changes than the original SNV-based annotation

The overall process of correcting MNV annotations occurs as two steps: 1) haplotype phasing, and 2) determining the protein codon (Fig. 2). MAC can be run with either ‘no-annotation’ mode or one of three pre-compiled genetic variant annotating tools (ANNOVAR, SnpEff and VEP). MAC will output explicit haplotype phasing information either way so that users have full flexibility in annotator selection. If the ‘no-annotation’ mode is specified, MAC will output any identified BMs by reporting haplotype phase information and the corresponding read counts. The user can then select their own annotating tool to annotate each haplotype, and fix any co-located SNVs with overlapping codons (Fig. 2). If the user chooses to use one of the three pre-compiled annotators, MAC will incorporate the codon information with the haplotype phase to identify SNVs with overlapping codons within each BM. Therefore, our motivation for not using a simple codon-based screening approach is to provide explicit haplotype phasing information so that the users have more flexibility in annotator selection.

### Output

The report of MAC is based on the identified BMCs. Each BMC may contain multiple haplotypes and each row corresponds to one haplotype in a certain BMC. The following columns are included: 1) BMC ID consisting of all SNVs in the BMC; 2) an index number of the haplotype in current BMC; 3) the status of each SNV in the current haplotype: mutant, non-mutant, or unknown; 4) the number of unique NGS reads supporting current haplotype; 5) corrected annotation including gene, mRNA transcript and amino acid change for any haplotype containing at least one SNVs.

## Results

To evaluate the MAC software, a test data set containing 3024 somatic SNVs detected by whole genome sequencing in breast cancer specimens (Wei et al., in preparation) was used (Fig. 2). After initial sequencing read extraction and grouping, 56 *Block of Mutations* (BMs) were identified containing 129 total SNVs. The sizes of the 56 BMs, in terms of the numbers of SNVs, range from 2 SNVs (44), 3 SNVs (9), 4 SNVs (2) to 6 SNVs (1). Upon manual review, the BM with 6 SNVs is located near a structural variation and appears to fit the previously described phenomenon of “Kataegis” [13], a pattern of confined hypermutation co-localized with structural variations in cancer (data not shown). A total of 4 BMCs were identified after selecting for SNVs with overlapping codons. All 4 BMCs are dinucleotide variations, and a mini BAM file containing all these 4 BMCs is available in the MAC package. A detailed comparison for the 4 identified dinucleotide variations shows that the MNV-based amino acid change prediction is different from the amino acid change predicted in the SNV-based set (Table 1). Specifically, 7 of the 8 SNVs were predicted to cause different amino acid change after re-annotation. In one particular case of ZNF407, a missense MNV was annotated as nonsense in the previous SNV-based annotation.

**Table 1:** Results of SNV- and MNV- based amino acid predictions in test MAC run

	Mutation	SNV annotation^a^	MNV annotation	Gene (mRNA)
1	chr6.12121325.C > G	P433A (missense)	P433G (missense)	HIVEP1 (NM_002114)
2	chr6.12121326.C > G	P433R (missense)		
3	chr17.7577084.T > A	E285V (missense)	E285L (missense)	TP53 (NM_000546)
4	chr17.7577085.C > G	E285Q (missense)		
5	chr6.44376224.C > G	A316G (missense)	A316G (missense)	CDC5L (NM_001253)
6	chr6.44376225.G > C	A316A (silent)		
7	chr18.72775594.T > A	L1973I (missense)	L1973K (missense)	ZNF407 (NM_017757)
8	chr18.72775595.T > A	L1973^a^ (nonsense)		

^a^The underscores indicate difference between SNV- and MNV- annotations

One of these 4 identified MNVs resides in the tumor suppressor gene TP53. The two SNVs occur at consecutive base pairs (chr17.7577084 and chr17.7577085), both in codon 285 glutamic acid (E) in TP53 (NM_000546) (Fig. 1, A1). The first SNV (chr17.7577084) was predicted to replace glutamic acid (E) by valine (V), while the second SNV (chr17.7577085) was predicted as a change to glutamine (Q) (Fig. 1, A2). However, neither of these two predictions was correct because these two mutations are co-located on the same chromosome. The actual amino acid product, after translating the two SNVs simultaneously, is leucine (L) (Fig. 1, A3). Therefore, to accurately assess the functional change of this TP53 mutation and others, it is important to determine the exact haplotype prior to annotating the amino acid change.

## Conclusions

We developed MAC, a program to support users of SNV-based callers to restore potentially incorrect amino acid change predictions by MNVs. The current approach employed by most NGS variant pipelines, *i.e.* treating a MNV as unrelated SNVs and annotating each variant separately, often leads to inaccurate results. Despite the overall low prevalence in most cancers, MNVs can happen at much higher frequency in certain cancer types. Accurate MNV annotation is especially important for correctly understanding the tumorigenic mechanisms in such cancers. Correcting these annotation errors requires haplotype phase information, which is not retained by most variant callers. Our program MAC solves the problem by retrieving the haplotype phase information from the BAM file, identifying and fixing any potentially mis-annotated protein codons. As the amount of sequencing data grows rapidly, this automated pipeline provides a convenient solution to bridge the gap between the commonly used SNV-based variant callers and the need for correct MNV annotation. Although the development of MAC was motivated by more frequently observed dinucleotide substitutions, MAC can also provide annotation correction for trinucleotide or other complex substitution mutations.

We foresee a variety of extensions to the applications of MAC. In addition to known cancer types with a high frequency of MNVs, MAC can provide accurate annotation for any genomic locations involving multiple haplotypes such as mutation hot spots. When running under the ‘no-annotation’ mode, MAC can also provide haplotype phase information for neighboring SNVs. This function can be useful in determining biallelic mutant status or characterizing tumor heterogeneity.

## Methods

The study was approved by Institutional Research Board (IRB) of the Roswell Park Cancer Institute (RPCI).

### Extracting reads from the BAM file for all SNVs to identify *Block of Mutations* (BM)

Starting with a list of SNVs previously identified by any variant caller, all SNVs are screened for the existence of MNVs and reclassified as such. The fact that two SNVs occur in the same protein codon does not imply that the mutant alleles fall on the same chromosome. Without knowing the haplotype phase information, MNVs (Fig. 1, A1) can be easily confused with neighboring SNVs from separate chromosomes (Fig. 1, B1). In order to correctly predict amino acid change, it is critical to distinguish the actual relationship by interrogating the raw NGS reads spanning neighbouring SNVs. NGS assays typically generate millions to billions of reads per sample, where each pair of reads (if paired-end sequencing) originates from the same DNA fragment. Therefore, the fact that two SNVs are consistently present on the same read or read pair implies that these two SNVs are on the same chromosome, and vice versa. In normal diploid genomes, there usually exists no more than two haplotypes, while more patterns can be observed in tumor samples due to tumor heterogeneity and chromosomal aneuploidy. Due to the short length of NGS reads, the relationship between any two random SNVs around the genome is usually difficult to determine. However, when the two SNVs occur close enough that the distance is less than the library insert size, haplotypes can be inferred by using the reads or read pairs spanning multiple SNVs (Fig. 1, A1 and B1).

To identify and utilize reads spanning multiple SNVs, we first treat every SNV as a vertex (Fig. 2, A1), then extract reads at every SNV’s site from the BAM file, and classify each read’s mutation status regarding the specific SNV into one of three groups: mutant, non-mutant (wild-type or a different allele), or unknown (ie. no coverage). Additionally, reads with a base quality lower than a specifiable quality threshold at a SNV position are classified as ‘unknown’ for the corresponding SNV. To maximize the chance of determining the relationship between multiple SNVs, we combined the mutation statuses of every pair of reads, which expands the length of each measurement from one read (usually 100 bp) to the size of the DNA fragment (named as insert, usually spans several hundred bps). To avoid potential sequencing errors, any read pair containing a controversial mutation status regarding one SNV (i.e. one read suggests mutant while its read pair suggests a non-mutant) will be excluded. For a simple description, the term “read” is used in this paper although we used read pair in the program.

A depth-first search strategy was implemented to screen through all the SNVs and their associated reads to find *Block of Mutations* (BM), defined as a group of mutations where every mutation contains at least one read or read pair (either mutant or non-mutant) that is shared with at least one other mutation in that group (Fig. 2, A2).

### Find *Block of Mutations within Codon* (BMC) and predict amino acid change by using a user-specified annotation tool

In certain circumstances, especially samples with high mutation burdens, one BM may contain many SNVs spanning several codons. This leads to a complex output as a significant number of different haplotypes can hypothetically result from the numerous combinations of multiple SNV mutations. Since our primary goal is to resolve the incorrect amino acid prediction of MNVs, we simplified the annotation process by limiting our focus to individual codons, thus annotating only one codon at one time.

To incorporate codon information, each BM was treated as a subgraph, and a second layer of edges (red in Fig. 2, A3) was added to any SNV with an overlapping codon. We defined any connected component by the second layer of edges (overlapping codon) in each subgraph as *Blocks of Mutations within Codon* (BMC). Those SNVs are processed together in the next annotation step. In situations when a block substitution spans across an intron/exon boarder, the non-coding part is excluded from subsequent functional prediction.

All existing haplotypes within each BMC were summarized with quantitative information. The sequencing reads are grouped by the combined mutation status of all SNVs. Specifically, within every haplotype, 1, 0, and dash (-) are used to represent the three possible mutation statues (mutant, non-mutant, and unknown, respectively) for each SNV. For example, a typical dinucleotide mutation on a single chromosome will be reported as 11, while two consecutive SNVs occurring separately on each chromosome pair will be reported as “10” or “01”. The numbers of reads with every haplotype are reported to provide relative abundance information.

In each haplotype, the existing variants were reformatted into one single block substitution and annotated with the selected annotation tool. In the final output, the predicted amino acid change for every existing haplotype is reported. Any haplotype containing an unknown SNV status will bypass the annotation process and not be reported, but MAC has the option to include these in the output without annotation, only reporting the corresponding read counts.

## Performance evaluation

To evaluate the performance of MAC, 10 lung squamous cell carcinomas (LUSC) samples with the highest level of somatic mutations as determined by whole exome sequencing, were selected from 178 TCGA (The Cancer Genome Atlas) LUSC tumor samples (https://tcga-data.nci.nih.gov/tcga/dataAccessMatrix.htm). The number of somatic mutations ranged from 743 to 3910. The corresponding TCGA bam files (GRCh37/HG19 assembly) were downloaded from Cancer Genomics Hub at https://browser.cghub.ucsc.edu. MAC was performed on each sample with the following annotation options: no-annotation, annotation-by-Annovar, annotation-by-VEP, and annotation-by-Snpeff (Table 2). The run times for all jobs were under 11 min. The peak memory usage when using the ‘no-annotation’ mode ranged from 380 MB to 1.2 GB, with a moderate positive correlation with the numbers of input SNVs (*r* = 0.6339). When running MAC with any pre-compiled annotator, most jobs had approximately the same peak memory usage across samples (Annovar: ~ 5 GB, VEP: 5-7 GB, Snpeff: ~ 13 GB). All jobs were performed on cluster nodes containing 16 cores with CPU of 2.60GHz (Model: Intel(R) Xeon(R) CPU E5-2670) and memory size of 64 GB. The operating system was CentOS release 5.7 (Final).

**Table 2:** Performance evaluation of MAC on 10 TCGA tumor samples (LUSC)

Sample barcode	Num. of input SNVs	Num. of BMs	Size of the largest BM	No Annotation		Annovar			VEP			Snpeff		
				Memory (Kb)	Run time (min:sec)	Num. of BMCs	Memory (Kb)	Run time (min:sec)	Num. of BMCs	Memory (Kb)	Run time (min:sec)	Num. of BMCs	Memory (Kb)	Run time (min:sec)
TCGA-18-3409-01A-01D-0983-08	3910	303	4	1123088	02:18.1	108	5185632	01:47.5	108	6701680	10:08.5	112	13766144	05:00.4
TCGA-22-5473-01A-01D-1632-08	944	21	3	652336	01:05.9	6	5180416	01:10.1	6	5017200	02:35.5	6	13654800	04:10.1
TCGA-33-4566-01A-01D-1441-08	1451	41	3	844304	01:23.5	18	5180864	01:29.0	20	5020896	03:20.2	19	13706992	04:27.2
TCGA-34-5231-01A-21D-1817-08	743	15	4	459488	00:53.8	7	5180416	00:58.6	7	5015952	02:12.6	7	13635536	03:55.1
TCGA-37-5819-01A-01D-1632-08	839	31	2	380144	00:41.0	10	5184032	00:44.1	11	5013136	02:29.6	12	13725376	03:55.3
TCGA-39-5031-01A-01D-1441-08	754	22	3	549296	00:52.5	1	5180384	00:59.2	1	5018256	02:21.9	1	13644784	03:58.5
TCGA-46-3769-01A-01D-0983-08	1037	29	3	446448	00:41.8	13	5184032	01:04.3	13	5025968	02:26.0	13	13639456	03:49.8
TCGA-60-2698-01A-01D-1522-08	1396	34	5	1198992	02:07.6	4	5180400	02:07.2	4	5021424	03:33.8	4	13715488	04:54.1
TCGA-66-2785-01A-01D-1522-08	1338	44	3	1101216	01:56.5	4	5180848	01:54.0	4	5025424	03:33.9	4	13731600	04:53.6
TCGA-85-6561-01A-11D-1817-08	984	14	2	602112	00:44.0	3	5180400	01:31.6	3	5016176	02:26.3	3	13637520	04:09.9

Num. of input SNVs: the number of somatic SNVs from TCGA data matrix

Num. of BMs: the number of identified *Blocks of Mutations*

Size of the largest BM: the maximu number of SNVs in a *Block of Mutations*

Num. of BMCs: the number of BMCs (*Block of Mutations wtihin Codon*)

Memory: the peak memory used during the run

Run time: the eclipsed wall time for the run

## Availability and requirements

**Project name:** MNV Annotation Corrector

**Project home page:**http://sourceforge.net/projects/mnvannotationcorrector/or https://github.com/hubentu/MAC

**Operating system (s):** Unix-like (Linux, Mac OSX)

**Programming language:** Perl

**Any restrictions to use by non-academics:** None
